# Variations in neonatal mortality, infant mortality, preterm birth and birth weight in England and Wales according to ethnicity and maternal country or region of birth: an analysis of linked national data from 2006 to 2012

**DOI:** 10.1136/jech-2019-213093

**Published:** 2020-01-21

**Authors:** Charles Opondo, Hiranthi Jayaweera, Jennifer Hollowell, Yangmei Li, Jennifer J Kurinczuk, Maria A Quigley

**Affiliations:** 1 NIHR Policy Research Unit in Maternal Health and Care, National Perinatal Epidemiology Unit, Nuffield Department of Population Health, University of Oxford, Oxford, UK; 2 School of Anthropology, University of Oxford, Oxford, UK

**Keywords:** birth weight, child health, cohort studies, epidemiology, infant mortality

## Abstract

**Background:**

Risks of adverse birth outcomes in England and Wales are relatively low but vary across ethnic groups. We aimed to explore the role of mother’s country of birth on birth outcomes across ethnic groups using a large population-based linked data set.

**Methods:**

We used a cohort of 4.6 million singleton live births in England and Wales to estimate relative risks of neonatal mortality, infant mortality and preterm birth, and differences in birth weight, comparing infants of UK-born mothers to infants whose mothers were born in their countries or regions of ethnic origin, or elsewhere.

**Results:**

The crude neonatal and infant death risks were 2.1 and 3.2 per 1000, respectively, the crude preterm birth risk was 5.6% and the crude mean birth weight was 3.36 kg. Pooling across all ethnic groups, infants of mothers born in their countries or regions of ethnic origin had lower adjusted risks of death and preterm birth, and higher gestational age-adjusted mean birth weights than those of UK-born mothers. White British infants of non-UK-born mothers had slightly lower gestational age-adjusted mean birth weights than White British infants of UK-born mothers (mean difference −3 g, 95% CI −5 g to −0.3 g). Pakistani infants of Pakistan-born mothers had lower adjusted risks of neonatal death (adjusted risk ratio (aRR) 0.84, 95% CI 0.72 to 0.98), infant death (aRR 0.84, 95% CI 0.75 to 0.94) and preterm birth (aRR 0.85, 95% CI 0.82 to 0.88) than Pakistani infants of UK-born Pakistani mothers. Indian infants of India-born mothers had lower adjusted preterm birth risk (aRR 0.91, 95% CI 0.87 to 0.96) than Indian infants of UK-born Indian mothers. There was no evidence of a difference by mother’s country of birth in risk of birth outcomes among Black infants, except Black Caribbean infants of mothers born in neither the UK nor their region of origin, who had higher neonatal death risks (aRR 1.71, 95% CI 1.06 to 2.76).

**Conclusion:**

This study highlights evidence of better birth outcomes among UK-born infants of non-UK-born minority ethnic group mothers, and could inform the design of future interventions to reduce the risks of adverse birth outcomes through improved targeting of at-risk groups.

## Introduction

Disparities in birth outcomes across ethnic groups persist in high-income countries, with minority ethnic group infants experiencing poorer birth outcomes than those of majority groups.^1–3^ In England and Wales, among babies born in 2014 and 2015 and infant mortality rates of Pakistani, Black Caribbean and Black African babies were more than double those of White non-British babies and more than one-and-a-half-times those of White British babies.^4^ While the overall risks of adverse birth outcomes in high-income countries are already low relative to global averages, to achieve further reductions, interventions targeted at reducing disparities in outcomes across ethnic groups will be required.^5^

In the UK, increasing proportions of births occur to women born outside the UK. In 2017, 28.4% of births occurred among women who were born outside the UK,^6^ compared with 26.5% in 2013.^7^ There are similar trends in other high-income countries: in the USA, the proportion of births to migrant women is projected to rise from 13.3% in 2014 to 18.2% by mid-century.^8^ In the European Union (EU), in 2011 the total fertility rate among women born in the EU was 1.70 compared with 1.88 among women born outside the EU,^9^ and 14% of births in 2013 were to women born outside the EU.^10^ These demographic changes, as well as changes in migration patterns, such as the large and, in recent years, increasing proportion of non-European migration to the UK,^11^ have important implications for future trends in birth outcomes considering the disproportionate burden of adverse birth outcomes among individuals belonging to minority ethnic groups, many of whom are foreign-born.

Much of the research on migration and health outcomes focuses on comparing the birth outcomes of migrant women to those of the native women.^12^ However, there is emerging evidence that even within ethnic groups, mother’s country of birth may influence birth outcomes. For example in the USA, the infants of migrant Hispanic women have lower risks of preterm birth, low birth weight and small-for-gestational-age than infants of Hispanic women born in the USA.^13^ Similarly, the infants of migrant non-Hispanic black women have lower risks of preterm birth and small-for-gestational-age than infants of non-Hispanic Black US-born women.^14^ In the UK, lower risks of adverse birth outcomes have been documented in infants of migrant minority ethnic group mothers compared with infants of UK-born minority ethnic mothers.^15 16^ However, these studies have focused on very broadly defined groups of the ethnic minority population, for example, by aggregating individuals of south Asian^15^ or of African and Caribbean^16^ origin into single groups.

With the availability of national linked data that includes ethnic groups of babies, mothers’ country of birth and birth outcomes for all babies born in England and Wales since 2005,^17 18^ there is now scope to further explore the role of mother’s county or region of birth on birth outcomes across more ethnic groups than was previously possible. Our aim was to investigate relative risks of neonatal mortality, infant mortality, preterm birth and differences in birth weights, contrasting between infants of mothers who were born in the UK, in their countries or regions of ethnic origin or elsewhere, and in particular using analyses stratified by ethnic group to identify any ethnic group-specific effects.

## Methods

### Study design

We analysed data from the cohort of all singleton live births occurring between 22 and 43 weeks of gestation in England and Wales from 1^st^ January 2006 to 31^st^ December 2012.

### Data

Statutory birth and death registration data for England and Wales were linked to the National Health Service Numbers for Babies (NN4B) birth notifications system by the Office for National Statistics (ONS). The NN4B system included additional characteristics such as ethnic group of babies and gestational age at birth, which were not collected at birth registration. The data linkage and its evaluation have been described elsewhere.^18 19^ Observations with birth weight exceeding twice the IQR above or below the median within each sex-gestation-ethnic group stratum, and those with gestational age greater than 43 weeks, were removed from the data set as these values were deemed implausible. Observations with missing birth weight were also removed, as it was not possible to evaluate whether they met the criteria for inclusion.

### Variables

Birth outcomes of interest were neonatal death, infant death, preterm birth and birth weight. Neonatal death was defined as any death within the first 28 days after birth, infant death was defined as any death within the first year after birth (including neonatal death) and preterm birth was defined as a birth before 37 completed weeks of gestation.

Mother’s country of birth was the main explanatory variable. It was coded from the National Statistics Category Classification title of the country of birth reported in the birth registration data. Mother’s country of birth was further subclassified within categories of infant’s ethnicity as described below. Infant’s ethnicity, reported in the NN4B system^17^ according to the ethnic categories used in the 2001 Census in England and Wales, was recoded for analysis into: White British, White (other), Indian, Pakistani, Bangladeshi, Black Caribbean, Black African, ‘Mixed or Other’ (including: all mixed groups; ‘other’ Asian groups, that is, excluding those discretely categorised as Bangladeshi, Indian or Pakistani; other Black groups than those discretely categorised as Black African and Black Caribbean; Chinese and groups recorded as ‘other’) and a ‘not stated’ group. Mother’s ethnicity was not reported, but for the purposes of subclassifying mother’s country of birth, it was assumed to be the same as the infant’s provided that the infant was not of mixed or ‘other’ ethnicity. Based on the mother’s ethnicity and the country or region of origin of that ethnicity, mother’s country of birth was then classified into three categories: ‘UK’, ‘mother’s country or region of ethnic origin’ or ‘elsewhere’ if born in neither the UK nor the country or region of origin of their ethnic group. Some ethnic groups — Indian, Pakistani and Bangladeshi — were generally consistent with a single country of ethnic origin, India, Pakistan and Bangladesh, respectively, but others, for example Black Caribbean and Black African (excluding North African), implied ethnic origins from a region of the world rather than a single country. Mothers of infants in the White (other) ethnic group were classified as born in their region of ethnic origin if they were born in the rest of Europe, North, Central and South America, Australia and New Zealand. It was not possible to correctly classify the reported countries of birth of non-UK-born mothers of babies whose ethnicities were reported as ‘Mixed/other’ or ‘Not stated’; they were therefore excluded from the main analysis of the effect of mother’s country of birth. However, the ‘Not stated’ group were included in a sensitivity analysis in which they were assumed to be White British, given previous findings of a similarity in characteristics between them and the White British infants.^20^ For White British mothers, country of birth was only classifiable as ‘UK’ or ‘elsewhere’ given that their country or region of birth was the UK.

We considered the potential for confounding by maternal and infant characteristics, year of birth and geopolitical circumstances of different countries and regions. The variables representing these factors in the data set were: maternal age, sex of infant, birth registration type (whether by married parents, unmarried parents living at the same address, unmarried parents living at different addresses or a sole registrant), infant’s year of birth, area-based deprivation measured using the index of multiple deprivation (IMD) — based on the mother’s address — and whether the mother’s country of birth was considered a fragile state based on the World Bank ‘Low-income countries under stress’ and ‘fragile situation’ classifications.^21^

### Analysis

We calculated the absolute risks of neonatal death, infant death and preterm birth, and the mean birth weights across characteristics and overall. Crude and adjusted risk ratios for neonatal death, infant death and preterm birth were estimated using binomial regression, and the mean differences in gestational age-adjusted birth weight were estimated using linear regression, with UK-born groups as the reference category. We adjusted for maternal age (including a quadratic term for age to account for departures from linearity), birth registration type, year of birth, sex of infant, IMD decile and for Black African infants only, fragile state status of mother’s country of birth. Gestational age was fitted as a continuous variable in models that adjusted for it. The robust estimator of variance was used to allow for non-independence of birth outcomes among infants born to the same mother. All regression models were fitted separately for each ethnic group although pooled estimates and tests for effect modification by ethnic group are also reported. The analysis was conducted using Stata V.15.

## Results

### Cohort characteristics

There were data on 4 744 666 infants in the linked data set provided by the ONS. After excluding 109 734 infants with implausible or missing birth weights or gestational ages, a total of 4 634 932 infants were eligible for inclusion in this analysis^19^; their characteristics are summarised in table 1.

**Table 1:** Characteristics of the infants in the data set

		Neonatal deaths		Infant deaths		Preterm births		Birth weight	
		N	Per 1000*	N	Per 1000*	N	%	Mean, kg	SD
Infant ethnic group, n (%)									
White British	3 009 231 (64.9)	5554	1.8	8634	2.9	166 663	5.5	3.40	0.55
White (other)	340 526 (7.4)	555	1.6	838	2.5	15 747	4.6	3.41	0.51
Indian	132 651 (2.9)	317	2.4	473	3.6	7984	6.0	3.10	0.51
Pakistani	180 269 (3.9)	728	4.0	1247	6.9	10 813	6.0	3.14	0.52
Bangladeshi	62 948 (1.4)	170	2.7	277	4.4	3964	6.3	3.08	0.50
Black Caribbean	47 505 (1.0)	190	4.0	285	6.0	3900	8.2	3.17	0.58
Black African	154 076 (3.3)	520	3.4	797	5.2	9534	6.2	3.30	0.57
Mixed or other	419 970 (9.1)	901	2.1	1431	3.4	23 678	5.6	3.29	0.53
Not stated	287 756 (6.2)	703	2.4	1019	3.5	16 232	5.6	3.36	0.55
Sex of infant, n (%)									
Male	2 377 766 (51.3)	5431	2.3	8464	3.6	140 994	5.9	3.42	0.56
Female	2 257 166 (48.7)	4207	1.9	6536	2.9	117 521	5.2	3.30	0.53
Year of birth, n (%)									
2006	631 705 (13.6)	1521	2.4	2303	3.6	37 858	6.0	3.35	0.56
2007	646 902 (14.0)	1493	2.3	2383	3.7	37 184	5.8	3.36	0.55
2008	663 918 (14.3)	1427	2.1	2241	3.4	37 124	5.6	3.36	0.55
2009	659 807 (14.2)	1371	2.1	2130	3.2	36 687	5.6	3.36	0.55
2010	671 265 (14.5)	1314	2.0	2042	3.0	36 157	5.4	3.37	0.54
2011	675 075 (14.6)	1323	2.0	2033	3.0	36 472	5.4	3.37	0.54
2012	686 260 (14.8)	1189	1.7	1869	2.7	37 033	5.4	3.37	0.54
Mother’s age in years, mean (SD)	29.0 (6.0)								
Mother’s country or region of birth†, n (%)									
UK	3 507 324 (75.7)	7033	2.0	10 955	3.1	199 357	5.7	3.38	0.55
Country or region of ethnic origin	622 475 (13.4)	1599	2.6	2516	4.0	33 728	5.4	3.29	0.54
Elsewhere	505 133 (10.9)	1006	2.0	1530	3.0	25 430	5.0	3.33	0.53
Birth registration type, n (%)									
Married parents	2 499 063 (53.9)	4760	1.9	7210	2.9	124 912	5.0	3.38	0.54
Joint registration, same address	1 398 935 (30.2)	2945	2.1	4433	3.2	79 657	5.7	3.37	0.55
Joint registration, different addresses	450 500 (9.7)	1216	2.7	2001	4.4	32 143	7.1	3.28	0.57
Sole registrant	286 434 (6.2)	717	2.5	1357	4.7	21 803	7.6	3.25	0.57
Index of multiple deprivation decile, n (%)									
1 – most deprived	662 767 (14.3)	1868	2.8	3098	4.7	44 384	6.7	3.27	0.56
2	598 259 (12.9)	1558	2.6	2468	4.1	37 615	6.3	3.30	0.56
3	541 795 (11.7)	1340	2.5	2066	3.8	32 461	6.0	3.33	0.55
4	489 932 (10.6)	1007	2.1	1599	3.3	27 819	5.7	3.35	0.55
5	439 270 (9.5)	823	1.9	1264	2.9	23 447	5.3	3.38	0.54
6	422 908 (9.1)	770	1.8	1144	2.7	21 862	5.2	3.40	0.54
7	388 665 (8.4)	629	1.6	941	2.4	19 586	5.0	3.41	0.53
8	382 585 (8.3)	607	1.6	893	2.3	18 540	4.9	3.42	0.53
9	366 851 (7.9)	550	1.5	822	2.2	17 421	4.8	3.43	0.52
10 – least deprived	341 900 (7.4)	486	1.4	706	2.1	15 380	4.5	3.45	0.52
**Total**	4 634 932	9638	**2.1**	15 001	**3.2**	258 515	**5.6**	**3.36**	**0.55**

*Mortality rates per 1000 live births.

†Re-coded from mother’s actual country of birth and classified according to mother’s ethnic group – see ‘Methods’.

The majority of infants were white (72.3%), either of British (64.9%) or other White ethnicities (7.4%). The remaining 27.7% infants included 12.5% of known minority ethnic groups, 9.1% mixed race infants and 6.2% infants whose ethnicity was not stated.

More than three quarters (75.7%) of infants had UK-born mothers, with the remaining quarter approximately equally split between mothers born in their countries or regions of ethnic origin and those born elsewhere. The average age of all mothers was 29 years. Most birth registrations (53.9%) were by married parents and nearly a third (30.2%) were by unmarried cohabiting parents. The distribution of infants across deciles of IMD was uneven, with more infants born in more deprived areas.

**Figure 1:**
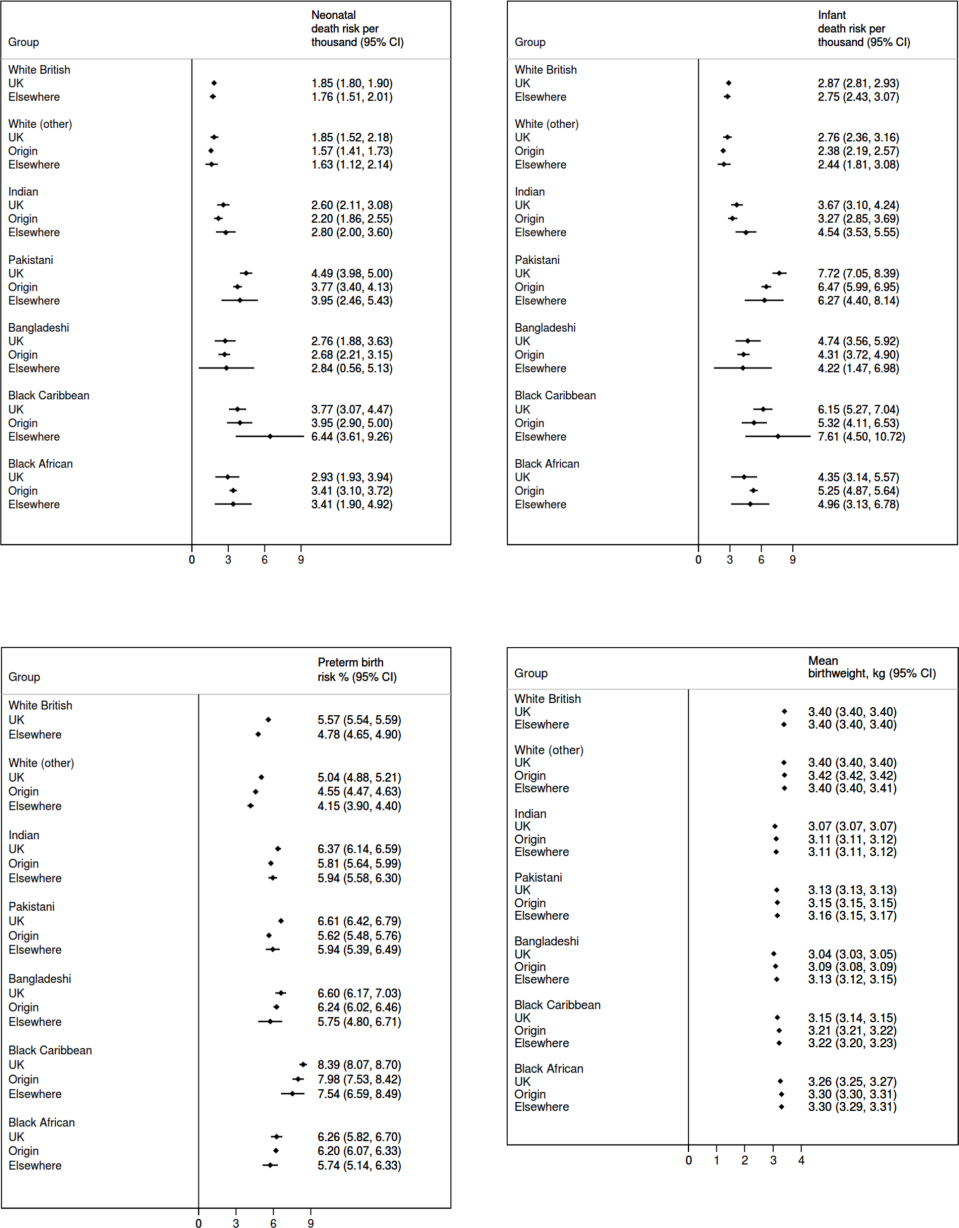
Absolute adjusted risks of neonatal death (top left), infant death (top right), preterm birth (bottom left) and mean gestational age-adjusted birth weight (bottom right) across ethnic groups by mother’s country or region of birth.

The highest absolute risks of neonatal and infant deaths were observed among Pakistani and Black Caribbean infants, and the highest absolute risks of preterm births were in Black Caribbean and Bangladeshi infants (table 1, figure 1). Bangladeshi, Indian and Pakistani infants had the lowest mean birth weights. White (other) infants had the lowest absolute risks of adverse birth outcomes and the highest mean birth weights, followed by White British infants.

Absolute risks of adverse outcomes and mean birth weight were lower among female infants compared with males, infants born in later years compared with the earlier ones, infants born to married parents compared with unmarried or single parents and infants born in less deprived areas compared with those born in more deprived areas.

### Mother’s country or region of birth

**Figure 2:**
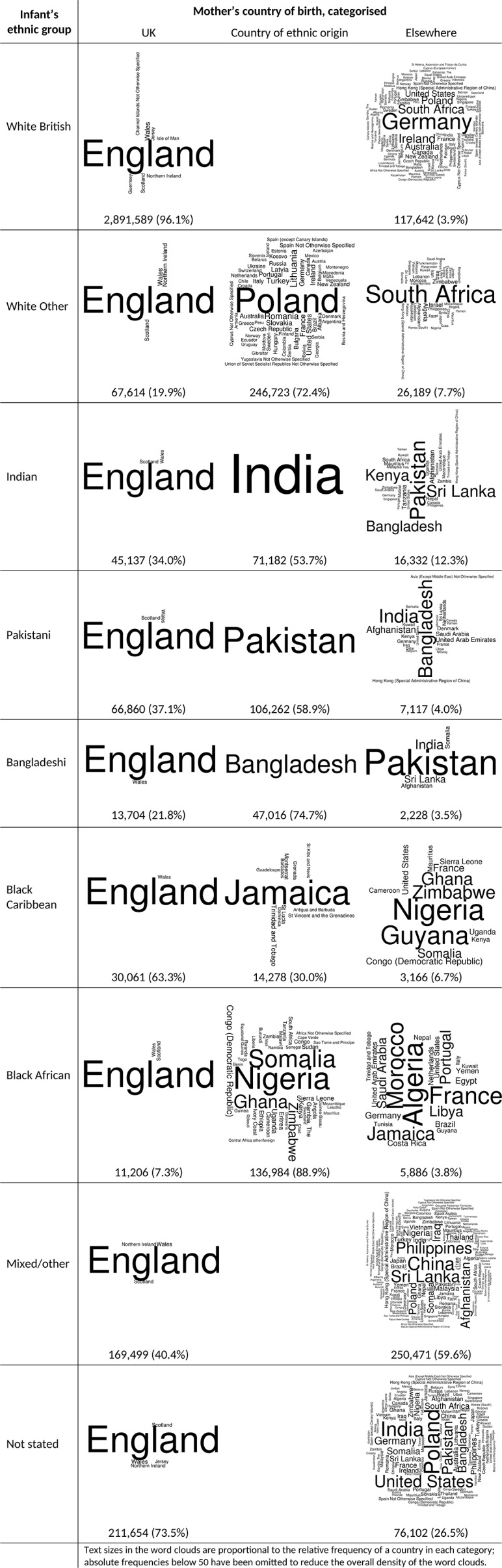
Mother’s country of birth classified according to ethnic group.

The majority of the mothers of White British infants (96.1%), Black Caribbean infants (63.3%) and infants whose ethnicity was not stated (73.5%) were born in the UK (figure 2). Between 53.7% and 88.9% of the mothers of White Other, Indian, Pakistani, Bangladeshi and Black African infants were born in places which were classified in these analyses as their countries or regions of ethnic origin. Only 3.8% to 12.3% of mothers were born ‘elsewhere’, that is, neither in the UK nor countries or regions considered to be of their ethnic origin. Among infants of mixed/other ethnicity and those whose ethnicity was not stated — for whom mother’s country or region of ethnic origin could not be determined and who were therefore excluded from further analysis — 59.6% and 26.5% of mothers, respectively, were classified as born ‘elsewhere’.

### Neonatal death

The overall risk of neonatal death was 2.1 per 1000, but it varied from 1.9 per 1000 in infants of UK-born mothers to 2.6 per 1000 in infants of mothers born in their countries or regions of ethnic origin (table 2). The highest absolute risk of neonatal death was observed among Pakistani and Black Caribbean infants, both groups having absolute risks of 4.0 per 1000, which was more than double the risk in the groups with the lowest risks, White British (1.8 per 1000) and White Other (1.6 per 1000) (table 1). In the pooled analysis, infants of mothers born in their countries of ethnic origin had higher crude risk of neonatal death than those of UK-born mothers (crude risk ratios (RRs) 1.32, 95% CI 1.25 to 1.39), but infants whose mothers were born elsewhere had similar crude risk of neonatal death as those of UK-born mothers (crude RRs 1.00, 95% CI 0.90 to 1.11) (table 3). Nevertheless the pooled adjusted risk of neonatal death among the infants of mothers born in their countries or regions of ethnic origin was 9% lower than that of the infants of UK-born mothers (adjusted RR 0.91, 95% CI 0.83 to 0.997). There was no evidence of a pooled adjusted difference in risk of neonatal death comparing infants of mothers born elsewhere to those of UK-born mothers.

**Table 2:** Means and proportions of outcomes by mother’s country or region of birth across ethnic groups of infants included in the analysis

Ethnicity of infant	Number of infants in group			Neonatal deaths per 1000			Infant deaths per 1000			Preterm births %			Birth weight, mean kg (SD)		
	UK	Origin	Elsewhere	UK	Origin	Elsewhere	UK	Origin	Elsewhere	UK	Origin	Elsewhere	UK	Origin	Elsewhere
White British	2 891 589	_	117 642	1.9	_	1.6	2.9	_	2.4	5.6	_	4.5	3.40 (0.55)	_	3.44 (0.52)
White (other)	67 614	246 723	26 189	1.9	1.6	1.5	2.8	2.4	2.2	5.3	4.5	3.9	3.38 (0.53)	3.42 (0.51)	3.42 (0.50)
Indian	45 137	71 182	16 322	2.5	2.2	2.9	3.5	3.3	4.7	6.4	5.7	6.2	3.07 (0.51)	3.12 (0.50)	3.11 (0.51)
Pakistani	66 860	106 292	7117	4.4	3.8	3.8	7.6	6.5	6.0	6.6	5.6	5.9	3.12 (0.53)	3.16 (0.52)	3.16 (0.52)
Bangladeshi	13 704	47 016	2228	2.8	2.7	2.7	4.6	4.4	4.0	6.5	6.3	6.0	3.03 (0.50)	3.09 (0.50)	3.16 (0.51)
Black Caribbean	30 061	14 278	3166	3.8	3.9	6.3	6.2	5.3	7.3	8.4	8.1	7.2	3.14 (0.58)	3.22 (0.58)	3.25 (0.59)
Black African	11 206	136 984	5886	3.0	3.4	3.4	4.6	5.2	4.9	6.8	6.2	5.7	3.25 (0.57)	3.30 (0.57)	3.32 (0.56)
**Total**	3 126 171	622 475	178 560	**1.9**	**2.6**	**1.9**	**3.0**	**4.0**	**2.9**	**5.6**	**5.4**	**4.8**	**3.39 (0.55**)	**3.29 (0.54**)	**3.38 (0.53**)

**Table 3:** Crude and adjusted associations between mother’s country of birth and neonatal death, infant death, preterm birth and birth weight, pooled across ethnic groups and stratified by ethnic group

Ethnicity of infant and mother’s country or region of birth	Neonatal death, RR (95% CI)		Infant death, RR (95% CI)		Preterm birth, RR (95% CI)		Birth weight difference, g (95% CI) *	
	Crude	Adjusted†	Crude	Adjusted†	Crude	Adjusted†	Crude	Adjusted†
**Pooled analysis**								
All ethnic groups‡								
UK	1	1	1	1	1	1	0	0
Region of ethnic origin	**1.32** (**1.25 to 1.39**)	**0.91** (**0.83 to 0.997**)	**1.33** (**1.27 to 1.39**)	**0.91** (**0.85 to 0.98**)	**0.96** (**0.95 to 0.97**)	**0.89** (**0.88 to 0.91**)	**-78** **(-79 to -77**)	**25** (**23 to 27**)
Elsewhere	1.00 (0.90 to 1.11)	0.98 (0.88 to 1.10)	0.96 (0.88 to 1.05)	0.97 (0.89 to 1.07)	**0.84** (**0.82 to 0.86**)	**0.86** (**0.85 to 0.88**)	**-3** **(-5 to -1**)	**6** (**4 to 8**)
**Stratified analysis**								
White British								
UK	1	1	1	1	1	1	0	0
Elsewhere	**0.86** (**0.74 to** **0.995**)	0.95 (0.82 to 1.10)	**0.84** (**0.75 to 0.94**)	0.96 (0.85 to 1.08)	**0.81** (**0.79 to 0.83**)	**0.86** (**0.84 to 0.88**)	**25** (**23 to 28**)	**-3** **(-5 to -0.3**)
White (other)								
UK	1	1	1	1	1	1	0	0
Region of ethnic origin	0.85 (0.69 to 1.04)	0.85 (0.69 to 1.05)	0.85 (0.72 to 1.00)	0.86 (0.73 to 1.02)	**0.85** (**0.82 to 0.88**)	**0.90** (**0.87 to 0.94**)	**22** (**19 to 26**)	**21** (**18 to 25**)
Elsewhere	0.80 (0.56 to 1.14)	0.88 (0.61 to 1.27)	0.79 (0.59 to 1.06)	0.89 (0.66 to 1.19)	**0.74** (**0.69 to 0.79**)	**0.82** (**0.77 to 0.88**)	**28** (**22 to 34**)	**6** (**1 to 12**)
Indian								
UK	1	1	1	1	1	1	0	0
Country of ethnic origin	0.88 (0.69 to 1.12)	0.85 (0.66 to 1.09)	0.91 (0.74 to 1.11)	0.89 (0.73 to 1.09)	**0.90** (**0.85 to 0.94**)	**0.91** (**0.87 to 0.96**)	**37** (**33 to 42**)	**42** (**38 to 47**)
Elsewhere	1.15 (0.82 to 1.61)	1.08 (0.77 to 1.52)	1.31 (1.00 to 1.71)	1.24 (0.94 to 1.62)	0.97 (0.90 to 1.04)	0.93 (0.87 to 1.001)	**36** (**29 to 44**)	**41** (**34 to 48**)
Pakistani								
UK	1	1	1	1	1	1	0	0
Country of ethnic origin	**0.86** (**0.74 to 0.99**)	**0.84** (**0.72 to 0.98**)	**0.86** (**0.77 to 0.97**)	**0.84** (**0.75 to 0.94**)	**0.85** (**0.82 to 0.89**)	**0.85** (**0.82 to 0.88**)	**27** (**24 to 31**)	**23** (**19 to 27**)
Elsewhere	0.85 (0.58 to 1.27)	0.88 (0.59 to 1.30)	0.80 (0.58 to 1.08)	0.81 (0.60 to 1.11)	**0.90** (**0.81 to 0.989**)	**0.90** (**0.82 to 0.991**)	**35** (**25 to 45**)	**29** (**19 to 38**)
Bangladeshi								
UK	1	1	1	1	1	1	0	0
Country of ethnic origin	0.97 (0.67 to 1.39)	0.97 (0.68 to 1.40)	0.95 (0.72 to 1.26)	0.91 (0.68 to 1.21)	0.97 (0.90 to 1.04)	0.95 (0.88 to 1.02)	**63** (**55 to 70**)	**50** (**42 to 57**)
Elsewhere	0.97 (0.41 to 2.29)	1.03 (0.43 to 2.46)	0.88 (0.44 to 1.76)	0.89 (0.44 to 1.79)	0.92 (0.77 to 1.10)	0.87 (0.73 to 1.04)	**114** (**96 to 132**)	**96** (**78 to 114**)
Black Caribbean								
UK	1	1	1	1	1	1	0	0
Region of ethnic origin	1.03 (0.75 to 1.42)	1.05 (0.76 to 1.45)	0.84 (0.65 to 1.10)	0.87 (0.66 to 1.13)	0.96 (0.90 to 1.02)	0.95 (0.89 to 1.02)	**83** (**74 to 91**)	**67** (**59 to 75**)
Elsewhere	**1.67** (**1.04 to 2.68**)	**1.71** (**1.06 to 2.76**)	1.17 (0.76 to 1.80)	1.24 (0.80 to 1.91)	**0.85** (**0.75 to 0.97**)	0.90 (0.79 to 1.03)	**100** (**85 to 115**)	**71** (**56 to 86**)
Black African								
UK	1	1	1	1	1	1	0	0
Region of ethnic origin	1.12 (0.79 to 1.59)	1.16 (0.81 to 1.66)	1.15 (0.87 to 1.53)	1.21 (0.90 to 1.61)	**0.91** (**0.84 to 0.97**)	0.99 (0.92 to 1.07)	**31** (**23 to 39**)	**45** (**36 to 53**)
Elsewhere	1.12 (0.65 to 1.94)	1.16 (0.67 to 2.02)	1.08 (0.69 to 1.71)	1.14 (0.72 to 1.80)	**0.84** (**0.75 to 0.96**)	0.92 (0.81 to 1.04)	**37** (**23 to 50**)	**37** (**24 to 50**)

Bold text highlights estimates whose CI exclude no difference with the comparison group.

*All differences are gestational age-adjusted.

†Adjusted for maternal age (including quadratic term for departure from linearity), birth registration type, year of birth, sex of baby, IMD decile and additionally for Black African babies, whether mother’s country of birth was classified as a fragile state based on the World Bank ‘Low-income countries under stress’ and ‘fragile situation’ classifications.

‡Except ‘mixed/other’ and ethnicity ‘not stated’ because it was not possible to correctly classify countries or regions of birth for non UK-born mothers.

In the ethnic group-stratified analysis, there was no evidence of a difference in risk of neonatal death among infants of non-UK-born mothers compared with UK-born mothers, except among Pakistani infants for whom the adjusted risk of neonatal death was 16% lower among the infants of Pakistani mothers born in Pakistan (adjusted RR 0.84, 95% CI 0.72 to 0.98) and Black Caribbean infants whose mothers were born elsewhere for whom the adjusted risk of neonatal death was 71% higher than infants whose mothers were born in the UK (adjusted RR 1.71, 95% CI 1.06 to 2.76).

### Infant death

The infant death risk was 3.2 per 1000 overall but 3.0 per 1000 among infants of mothers born in the UK or elsewhere, and 4.1 per 1000 among infants of mothers born in their countries or regions of ethnic origin (table 2). Pakistani and Black Caribbean infants had the highest absolute risks of infant death, 6.9 per 1000 and 6.0 per 1000, respectively, while White British and White Other infants had the lowest, 2.9 per 1000 and 2.5 per 1000, respectively (table 1). As with neonatal death, in the pooled analysis infants, whose mothers were born in their countries or regions of ethnic origin had higher crude risk of infant mortality than infants of UK-born mothers (crude RR 1.33, 95% CI 1.27 to 1.39) but those whose mothers were born elsewhere had similar crude risks (crude RR 0.96, 95% CI 0.88 to 1.05) (table 3). However, the pooled adjusted risk of infant death was 9% lower among infants of mothers born in their countries or regions of ethnic origin (adjusted RR 0.91, 95% CI 0.85 to 0.98) compared with infants of UK-born mothers.

When stratified by ethnic group there was no evidence of a difference in risk of infant death when comparing infants whose mothers were born in their countries or regions of ethnic origin to those whose mothers were born in the UK across all ethnic groups, except among Pakistani infants: the adjusted risk of infant death was 16% lower among infants of Pakistani mothers born in Pakistan compared to infants of Pakistani mothers born in the UK (adjusted RR 0.84, 95% CI 0.75 to 0.94).

### Preterm birth

The overall preterm birth risk was 5.6%, varying from 4.8% among infants of mothers born elsewhere, to 5.6% among infants of UK-born mothers (table 2). The absolute risk of preterm birth was highest among Black Caribbean (8.2%) and Bangladeshi (6.3%) infants, and lowest among White British (5.5%) and White Other (4.6%) infants (table 1). In the pooled analysis the crude risk of preterm birth was lower among infants whose mothers were born in their countries or regions of ethnic origin (crude RR 0.96, 95% CI 0.95 to 0.97) and those whose mothers were born elsewhere (crude RR 0.84, 95% CI 0.82 to 0.86) (table 3). In the pooled adjusted analysis, infants of mothers who were born in their countries or regions of ethnic origin had 11% lower risk of preterm birth (adjusted RR 0.89, 95% CI 0.88 to 0.91) and those of mothers born elsewhere had 14% lower risk of preterm birth (adjusted RR 0.86, 95% CI 0.85 to 0.88) compared with infants of UK-born mothers.

In the ethnic group-stratified analysis, White Other, Indian and Pakistani infants of mothers born in their countries or regions of ethnic origin had lower adjusted risks compared with those whose mothers were born in the UK (adjusted RR 0.90, 95% CI 0.87 to 0.94; 0.91, 95% CI 0.87 to 0.96 and 0.85, 95% CI 0.82 to 0.88, respectively). Additionally, White Other and Pakistani infants whose mothers were born elsewhere had lower adjusted risks of preterm birth (adjusted RR 0.82, 95% CI 0.77 to 0.88 and 0.90, 95% CI 0.82 to 0.991) than those of UK-born mothers.

### Birth weight

The overall mean birth weight was 3.36 kg, varying slightly from 3.28 kg among infants of mothers born in their countries or regions of ethnic origin, to 3.39 kg among infants of mothers born in both the UK and elsewhere (table 2). Bangladeshi, Indian and Pakistani infants had the lowest mean birth weights: 3.08 kg, 3.10 kg and 3.14 kg, respectively. In the pooled analysis, the mean gestational age-adjusted birth weight of infants whose mothers were born in their countries or regions of ethnic origin was 78 g lower (95% CI −79 g to −77 g) than infants of UK-born mothers, and there was a smaller difference in mean birth weights between infants of mothers born elsewhere and infants of UK-born mothers (crude difference −3 g, 95% CI −5 g to −1 g) (table 3). However, the pooled adjusted difference in gestational age-adjusted mean birth weight was 25 g *higher* in infants of mothers born in their countries or regions of ethnic origin (95% CI 23 g to 27 g) and 6 g higher in infants of mothers born elsewhere (95% CI 4 g to 8 g) compared with infants of UK-born mothers. The ethnic group-stratified analysis showed that infants of non-UK-born mothers in all ethnic groups had higher adjusted mean birth weights than those of UK-born mothers, except White British infants of non-UK-born mothers whose gestational age-adjusted mean birth weight was 3 g lower (95% CI −5 g to −0.3 g) than that of UK-born mothers.

### Sensitivity analysis

It has previously been suggested that the infants whose ethnicity was not reported were probably White infants.^17^ We observed that the risks of neonatal and infant death in this group of infants were higher than those of White British infants (2.4 vs 1.8 and 3.5 vs 2.9 per 1000, respectively), but their risk of preterm birth and mean birth weight were similar (5.6% vs 5.5% and 3.36 kg vs 3.40 kg, respectively). Repeating the adjusted ethnic group-stratified analysis with the infants whose ethnicity was not reported re-coded as White British showed no evidence of a difference in risk of neonatal (adjusted RR 1.06, 95% CI 0.95 to 1.18) or infant death (adjusted RR 1.06, 95% CI 0.97 to 1.16) but evidence of a lower risk of preterm birth (adjusted RR 0.91, 95% CI 0.89 to 0.92) comparing infants whose mothers were born elsewhere to those of UK-born mothers, consistent with the findings of the main analysis. There was also evidence of a much lower mean birth weight, relative to the main analysis, in infants of mothers born elsewhere compared with those of UK-born mothers (adjusted difference −31 g, 95% CI −33 g to −29 g).

## Discussion

We explored the variations in birth outcomes among infants of different ethnicities born in England and Wales between 2006 and 2012. We sought to determine the extent to which these variations in birth outcomes could have been explained by where the mothers of these infants were born. To achieve this we compared the outcomes between infants born to UK-born mothers, the majority and infants born to non-UK-born mothers, who made up about a quarter of the births in the data. We further distinguished non-UK-born mothers between mothers born in their country or region of ethnic origin and elsewhere. Such distinctions included, for example an Indian mother born in India, and an Indian mother born in Africa, both of whom then migrated to the UK and gave birth. Although we used ‘ethnic origin’ broadly for the purpose of classifying mother’s country or region of birth according to their ethnicity, we recognise that no single approach to this classification can fully encapsulate the nuances of ethnicity, nor of individuals’ perception of their own origins and heritage.

Our pooled adjusted analysis showed evidence that the infants of non-UK-born mothers who were born in their countries or regions of ethnic origin had lower risks of neonatal death, infant death and preterm birth and higher mean birth weights than the infants of mothers born in the UK. There was evidence of a lower risk of preterm birth, but not neonatal or infant death, in the infants of mothers who were born neither in the UK nor in their country or region of ethnic origin (ie, those born ‘elsewhere’), and evidence of a higher mean birth weight than in infants of UK-born mothers. The adjusted ethnic group-stratified analysis showed lower risks of adverse birth outcomes among the infants of non-UK-born mothers to be particularly evident among Indian (for preterm birth) and Pakistani infants (for all outcomes), a higher risk of neonatal death among Black Caribbean infants whose mothers were born elsewhere and a positive effect on birth weight in all ethnic groups except White British infants.

These findings are consistent with those of previous studies in England and Wales which found higher risks of adverse birth outcomes among minority ethnic infants relative to White British infants,^22^ and also with studies exploring the role of ethnicity in explaining adverse birth outcomes among migrant groups in Europe and the USA which found increased risks of low birth weight and preterm birth among Asian and Black migrant populations.^23^ Our findings are also consistent with studies which show that infants of Indian, Pakistani and Bangladeshi women who were born in the Indian subcontinent had higher mean birth weights than infants of the same ethnic group of women born in England and Wales,^15^ and that infants of Black African mothers born in Western or ‘middle’ Africa had higher mean birth weights than infants of the same ethnicities born in the UK.^16^ What this study adds is evidence, particularly for infants of Pakistani women, that the risks of neonatal death, infant death and — additionally for Indian women — preterm births may also be lower for infants of mothers born in the Indian subcontinent compared with UK-born mothers. Our study also provides evidence that the higher mean birth weights in infants of non-UK-born mothers may apply to all ethnic groups, except infants of White British mothers.

The finding of evidence of higher risk of neonatal death among Black Caribbean infants of mothers born elsewhere stood in contrast with the pattern of findings of better outcomes among infants of non-UK-born mothers. A closer inspection found that the mothers of 64.9% of the non-UK-born Black Caribbean infants were born in Africa. This would imply that 11.8% of the non-UK-born Black Caribbean population between 2006 and 2012 was born in Africa, when in fact 2011 census data showed that only 1.3% of this group were born in Africa.^24^ Therefore, other than a true effect, a possible alternative explanation for this finding is widespread cross-classification of ethnicity between Black African and Black Caribbean infants. Indeed a previous study involving cancer patients in England has found up to 20% discordance between self-reported and hospital-recorded ethnicity of many major ethnic groups.^25^ While ethnicity should be self-declared it likely that in some instances it is ascribed by hospital staff.

Mother’s country of birth may influence birth outcomes of infants through conditions and risk factors which may be common to migrant groups, for example, health behaviour, cultural, environmental, economical, social and lifestyle factors,^26^ and experiences of migration. The finding of health advantages among migrant populations relative to native populations may be considered an epidemiological paradox,^27^ in view of the possible socioeconomic disadvantages^28^ and poorer experience of health care^29^ of the former. A number of potentially complementary factors may explain this paradox,^30^ including: health selection among some immigrant groups, including the ‘healthy worker’ effect by which healthier, better educated individuals are more likely to successfully migrate in search of work;^31^ healthier lifestyles among immigrants from some societies (‘cultural buffering’) including lower prevalence of smoking,^32^ alcohol consumption^33^ and obesity and immigration policies at destination which may profile and select immigrants on the basis of their health status.^34^ Subsequent generations may lose these advantages as they adopt health behaviours similar to the native populations. However, even within ethnic groups, there is considerable variation in the demographic and socioeconomic influences that may explain the heterogeneity of effect of mother’s country of birth on birth outcomes in the ethnic group-stratified analysis.

This study was based on a large number of births, which allowed us to conduct an ethnic group-stratified analysis with reasonable power to identify important differences. Our data set was drawn from national birth and death registration data and linked to routine hospital data to obtain covariates and additional outcomes not available in registration data, with few exclusions. It is therefore representative of England and Wales and may also apply to other high-income countries in Europe and possibly other countries with comparable health systems, socio-demographic characteristics and ethnic minority population distribution. However, our data did not include information about important risk factors for infant mortality, such as maternal smoking,^35^ alcohol consumption,^36^ diet and health,^37^ parity,^38^ breastfeeding^39^ and use of health services in the perinatal period,^40^ which would have been important to adjust for. Furthermore, groups such as Black Africans and White (other) are likely to be quite heterogeneous, and there is a possibility of heterogeneity in the association between mother’s country of birth and outcomes within these groups. For Black Africans, we made an attempt to account for additional heterogeneity by controlling for fragile state status, an important consideration given its role in driving some of the migration out of Africa; this factor explained some of the variability in preterm birth but not any of the other birth outcomes. Lastly, although the NN4B system is supposed to record the baby’s ethnicity as reported by the mother, it seems plausible that in a small proportion of cases, the ethnicity recorded may have been that of the mother, or it may be that it was reported by a healthcare professional rather than the mother.^17^ Furthermore, ethnic group was not reported in 6.2% of the cohort, a group larger than each of the non-White ethnic groups. A sensitivity analysis assuming that these were most probably White British infants did not alter our conclusions about the effect of mother’s country or region of birth on the risks of neonatal death, infant death or preterm birth, but showed evidence of a much lower gestational age-adjusted mean birth weight among infants whose mothers were born elsewhere compared with those whose mothers were born in the UK than the difference observed in the main analysis.

## Conclusions

This study shows evidence of better outcomes among minority ethnic group infants whose mothers were born outside the UK compared with those born in the UK, and highlights the heterogeneity across ethnic groups in the effect of mother’s country of birth on infants’ birth outcomes. If current migration and demographic trends continue into the future, increasing proportions of births in England and Wales will occur among non-UK-born women, the majority of whom belong to minority ethnic groups, and increasing proportions of the population will be of non-White ethnicity. With ethnic minority groups experiencing poorer birth (and health) outcomes than majority groups, future reductions in the overall rates of adverse outcomes will require interventions that prioritise identification and targeting of at-risk groups. These findings will therefore inform risk-stratification strategies in public health interventions and policies aiming to improve birth outcomes.

**What is already known on this subject**

- There is a disproportionate risk of adverse outcomes among infants with a minority ethnic heritage in the UK. Risk factors for adverse birth outcomes are generally well understood and specific causes of poorer outcomes in some ethnic groups are also well established. However, there is a dearth of studies exploring the joint effects of mother’s country of birth and ethnic group on birth outcomes across the range of ethnic groups in the UK.

**What this study adds**

- We highlight evidence of better birth outcomes among UK-born infants of non-UK-born mothers: infants of mothers born in their countries or regions of ethnic origin have better birth outcomes than those of UK-born mothers, but there is no evidence of a difference between birth outcomes for infants of mothers born elsewhere and those of UK-born mothers in most ethnic groups. We also show evidence of considerable heterogeneity across ethnic groups in the effect of mother’s country of birth on birth outcomes, with higher gestational age-adjusted mean birth weights in infants of non-UK-born mothers compared to UK-born mothers across all ethnic groups except White British infants, but lower risks of preterm births only among White, Indian and Pakistani infants, and lower risks infant or preterm birth among Pakistani infants. These findings could inform future healthcare interventions to identify and prioritise at-risk groups.
